# Population immunity to the three serotypes of poliovirus post-interruption of wild poliovirus transmission in Nigeria

**DOI:** 10.1016/j.jve.2025.100615

**Published:** 2025-10-29

**Authors:** Marycelin Mandu Baba, Aisha Abba Kawu, Sadiya Alhaji Bukar, Musa Sundu Melton, Abdulwahab Mala, Ibrahim Salisu, Bamidele Soji Oderinde

**Affiliations:** a Department of Medical Laboratory Science, College of Medical Sciences, University of Maiduguri, P.M.B. 1069, Maiduguri, Nigeria; b African Field Epidemiology Network, Borno Field Office, Nigeria; c Borno State Primary Health Care Development Agency, Nigeria; d World Health Organization, Borno State Office, Nigeria

**Keywords:** Poliovirus, Neutralizing antibodies, Serotypes, Polio vaccine, Vaccination, Borno state, Nigeria

## Abstract

The presence of neutralizing antibodies is considered a surrogate marker of protection against the three serotypes of poliovirus. The need to use the Microneutralization test in assessing the neutralizing antibodies to the three serotypes of polioviruses among vaccinated children aged 1–15 years informed this study. Of 400 children tested, 309 (77.3 %), 253 (63.3 %), and 308 (77.0 %) had neutralizing antibodies against P1, P2, and P3, respectively. Only 191 (47.8 %) had neutralizing antibodies against P1P2P3 simultaneously, the global target. Whilst 91 (22.8 %) had no neutralizing antibodies against P1, these children were protected against P2 (23.0 %) and P3 (43.9 %). Similarly, 147 (36.8 %) children had no neutralizing antibodies against P2, but were protected against P1 (66.0 %) and P3 (65.3 %). Furthermore, 52(13 %), 51(12.8 %), and 52 (13.0 %) had no neutralizing antibodies against the combination of P1P3, P1P2, and P2P3, respectively. Only 34 (8.5 %) of the children had no nAb to any of the three serotypes. The optimal number of Polio vaccine doses for effective immunity varied depending on the serotype. Also, gender differences may favor the speed at which children achieve the target antibody titers. Higher antibody titers (1:1280) were observed for P2 and P3, with six of the children having a titer of 1:10240 for P3. The combination of supplementary immunization activities and routine immunization generated a robust immune response across all poliovirus serotypes, in contrast to each of the two. The administrative data and population immunity were not commensurate. New strategies to increase immunity against the P1P2P3 simultaneously in all age groups are urgently required.

## 1 Introduction

Although Nigeria is no longer endemic for wild Poliovirus (WPV) transmission since August 2020, persistent outbreaks of circulating Vaccine-derived polioviruses (cVDPVs), which are mutated forms of WPV, continue to occur. A confirmed case of cVDPV is considered an outbreak.^1^^,^^2^ Globally, cVDPV2 cases increased from 366 in 2019 to 1082 in 2020 and declined to 308 in 2023 and 359 in 2024.^3^^,^^4^ In 2024, eight cVDPV1 were obtained in acute flaccid paralysis cases in the Democratic Republic of Congo and Mozambique.^5^^,^^6^ These outbreaks continue to spread across Africa from the Atlantic to the Indian Ocean. For instance, of the 182 global cVDPV2 cases from acute Flaccid paralysis (AFP) in 2024, 85 (46 %) were reported in Nigeria.^5^ In Borno State, cVDPV cases rose from 2 in 2023 to 8 in 2024. Although the drivers of these outbreaks have not yet been fully elucidated, we speculate that poor quality and delayed polio outbreak response; low population immunity, declining gut immunity in young children to the type 2 virus after the switch from tOPV (serotypes1,2,3) to bOPV (serotype 1 and 3) for routine immunization in 2016; and insufficient routine immunization coverage are the possible contributors. Incidentally, these outbreaks have persisted in Nigeria despite many innovative strategies engaged to ensure that every child is effectively vaccinated.^7^, ^8^

Interestingly, Borno state conducted outbreak responses with nOPV2 in July, November, and December 2023, but during August campaigns, a combination of nOPVs and fIPV was used. Notably, the nOPV2 is modified to be more genetically stable than Sabin mOPV2 and less likely to be associated with the emergence of cVDPV2 in low-immunity settings.^6^^,^^9^^,^^10^ Fractional IPV was considered a more cost-effective strategy because of the constraint in the global supply of IPV and the absence of a substantial difference in seroconversion between three doses of fIPV and three doses of full-dose IPV.^11^ Incidentally, these outbreaks have persisted in Nigeria despite a series of innovative strategies to ensure that every child is effectively vaccinated.^7^, ^8^

According to previous reports, neutralizing antibodies are considered a surrogate marker of a protective immune response to the pathogen.^12^, ^13^, ^14^, ^15^, ^16^ Notably, circulating vaccine-derived poliovirus (cVDPVs) outbreaks occur when OPV-related strains undergo prolonged circulation in communities with very low immunity.^17^^,^^18^ According to the Borno State Primary Health Care Development Board, the administrative coverage for 2023 campaigns was 102 %, 102 %, 100 %, and 95 % for July, August, November, and December, respectively. Similarly, the Lot Quality Assessments (LQAs) in July, November, and December 2023 were 100 % each (Supplementary Table 2). It became necessary to ascertain whether the high vaccine coverage is commensurate with the immunity measured by the presence of neutralizing antibodies to PV serotypes. This study aims to assess the presence of neutralizing antibodies to the three serotypes of polioviruses among children aged 1–15 years in Bama and Maiduguri Metropolitan Council (MMC) LGAs, Borno State, Nigeria.

## 2 Methodology

### 2.1 Study area

**Fig. 1:**
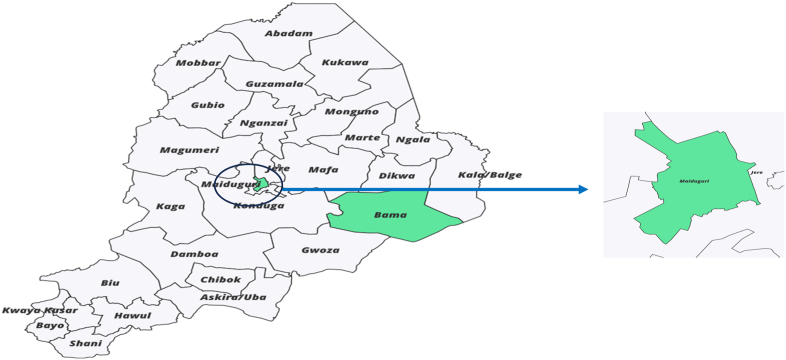
The map of Borno State showing the study sites in light green color Maiduguri is the capital and the largest city in Borno State in northeastern Nigeria. The city sits along the seasonal Ngadda River which disappears into the Firki swamps in the areas around Lake Chad. Maiduguri was founded in 1907 as a military outpost by the British. It is located at 11.85° latitude N|E and 13.16° longitude N|E and at an elevation 325 m above sea level.

The study was conducted in two local government areas (Maiduguri Metropolitan Council (MMC) and Bama) in Borno State, Nigeria (Fig. 1). Maiduguri is the capital and the largest city in Borno State, northeastern Nigeria. It is located at 11.85° latitude N|E and 13.16° longitude N|E with an estimated population of 870,000 in 2023.^19^ *Among all the LGAs in Borno, MMC is the most populated, with an estimated 2 million people, and is a center for many internally displaced persons (IDPs).* The hot season in Maiduguri lasts at least 2.4 months, from March to May, with an average daily high temperature above *38.9*^*o*^*C*^20^*. The hottest month of the year is usually May with an average high temperature of 39.4* °*C and a low of 26* °*C*^20^*.* Bama LGA is the second-largest town in Borno State, with an area of 4,997 km^2^ and a population of 269,986, according to the 2006 census. It shares borders with the Republic of Cameroon and is about 60 km (37 miles) from Maiduguri. Bama LGA coordinates on 11.5213^o^ latitude and 13.6895^o^ longitude. Bama was repeatedly attacked between May 2013 and September 2014 and finally captured by the insurgency. In 2018, the Borno state government approved the return of about 120,000 internally displaced persons (IDPs) to Bama town.

### 2.2 Ethical clearance and informed consent

Ethical clearance was obtained from the ethical committee of the Borno State Ministry of Health. The informed consent forms were distributed to the parents and/or guardians of children recruited for the study. Only children whose parents understood the type and purpose of the project and gave their consent were recruited for the study.

### 2.3 Study population

Healthy children aged between 1 and 15 years were recruited for the study (Table 1). This age group conforms to the AFP surveillance target group. Children who resided or attended schools located in Maiduguri and Bama were eligible. Inclusion criteria were extended to children who sought medical attention in any health facilities in Maiduguri as long as the reason for the medical attention was not connected or associated with the study. Only willing participants were consecutively enrolled in the study. Children with known immune disorders, or who received treatment with immunosuppressant drugs or radiotherapy in the previous 12 months, had received systemic corticoids in the last three months, or had received immunoglobulins or blood transfusions in the past three months, were excluded from the study. Brief, structured, closed-ended questionnaires were used to collect demographic data and vaccination history.

**Table 1:** Demographic characteristics of Subjects n = 400.

Variable	Category	Frequency	Percentage (%)
Gender	Male	224	56.0
	Female	176	44.0
	**Total**	**400**	**100.0**
Age	1–3	89	22.3
	4–6	82	20.5
	7–9	114	28.5
	10–12	67	16.8
	13–15	48	12.0
	**Total**	**400**	**100.0**
Type of immunization activity	RI	76	19.0
	SIA	289	72.3
	RT/SIA	35	8.8
	**Total**	**400**	**100**
**Local Government Area**	Bama	200	50.0
	MMC	200	50.0
	**Total**	**400**	**100.0**

Of 400 children tested, 224 were males and 176 were females. Their ages ranged from 1 to 15 years. The majority (289) of the children received Polio vaccines through Supplementary Immunization (SIA),Outbreak response (OBR), while only 76 were vaccinated through routine Immunization (RI), but only 35 were vaccinated through the combination of SIA and RI.

Sample Collection and Storage. 2–3 mL of blood was collected, allowed to clot, centrifuged, and the serum was aspirated into a sterile cryovial labeled with the biodata of each child. The serum samples collected within MMC were transported within 24 h to the World Health Organization National Polio Laboratory, Maiduguri, for storage and analysis. The samples collected from Bama were stored at −20 °C and were transported at 2–8 °C to the testing Laboratory, where all the samples were stored at −80 °C until tested.

### 2.4 Vaccination history of the children

The Expanded Programme on Immunization (EPI) was officially launched in Nigeria by the World Health Organization (WHO) in 1978, but the name was changed to the National Programme on Immunization in 1996. The programme aimed to provide immunization to children below the age of two years.^21^ Some strategies adopted by the Nigerian Government to improve immunization coverage include routine immunization (RI) intensification, supplemental immunization activities (SIAs), the use of global positioning system (GPS) trackers, emergency vaccination centres, and a range of community-level interventions.^22^^,^^23^ Additionally, Nigeria is also an active participant in the Global Vaccine Action Plan (GVAP), which aims to ensure equitable access to available vaccines worldwide.^24^

The children recruited for this study were born between 2008 and 2023. Within this period, all the children must have received different doses of polio vaccines, including tOPV, bOPV, mOPV2, and IPV. In 2014 and 2016, these children received the full dose of IPV. The vaccination history of the children was obtained either from RI cards or recall from the mothers/caregivers of the children who received the vaccine through SIA/OBR. Currently in Nigeria, polio Immunization campaigns are conducted using SIAs, outbreak response (OBR), and mop-up exercises targeting children under 5 years. The SIAs are intended to complement, not replace, routine immunizations. SIAs/OBR aim to interrupt the circulation of poliovirus by immunizing every child under five years of age with at least two doses of oral polio vaccine, regardless of previous immunization status. With this idea, children who are either not immunized or only partially protected are reached with the vaccine. These exercises also help to boost the immunity in those who have been immunized. Mop-up activities are based on clusters of unreached children that may have been missed in a specific geographic location.

The SIAs and OBR campaigns are in different forms, including well-coordinated house-to-house, special teams that visit churches, schools, mosques, and fixed teams (routine immunizations).

In 2023, when the samples of this study were collected, only nOPV2 and a combination of nOPV2 and fIPV were used for the outbreak response from July to December. (Supplementary Table 2). Notably, these children must have been exposed to vaccination with three serotypes of Poliovirus before 2023. Additionally, children vaccinated with the Oral polio vaccine can shed the vaccine strains in their stool, which can also vaccinate other non-vaccinated children.^25^, ^26^, ^27^

The Nigerian RI schedule includes a range of vaccines, including BCG, OPV, IPV, hepatitis B, DPT, measles, yellow fever, and pneumococcal vaccines (Supplementary Table 4). Precisely, children aged 0–24 h after birth usually receive OPV0, then OPV1 at 6 weeks, OPV2 at 10 weeks, and OPV3 at 14 weeks of age. Additionally, at six weeks and 14 weeks of age, IPV1 and IPV2 are usually administered, respectively. Overall, it is expected that children aged 12–23 months would have completed their immunizations and be fully immunized. Yet during campaigns, these children continue to receive the polio vaccine until they exceed five years of age.

**Manufacturers of the Polio vaccines used in Nigeria:** The bOPVs used for the vaccination exercises were manufactured by GSK Biologicals s.a, Rixensart, Belgium, nOPV2 by Bio Farma, a company based in Indonesia and IPV by Sanofi (France)/Sanofi (India), Bilthoven (Netherlands), AJ Vaccines (Denmark), and LG Chem (Korea).

### 2.5 Supply of the poliovirus reference strains

The reference strains Sabin1 (01/528) and Sabin 3 (01/532) were kindly supplied by the National Institute of Biological Standards (NIBSC), UK. In the absence of a reference strain for Poliovirus serotype 2, we used the Novel Oral Poliomyelitis Vaccine Type 2 (nOPV2), manufactured by B/E Biological E. Limited, Telegana, India-500078. The nOPV2 was supplied by the Epidemiological Unit (EPI) of the Borno State Ministry of Health, the central vaccine storage (−20 °C) facility in the State.

### 2.6 Preparation of the local virus stock (LVS) from the reference strains (poliovirus serotypes 1, 3) and nOPV2

The titration of the reference strains and nOPV2 was performed as described in the World Health Organization Polio Laboratory Manual.^28^ Preparation of the laboratory quality control Poliovirus serotypes 1, 2, and 3 stock was done from the reference strains supplied by NIBC in December 2018, and nOPV2 obtained from the Epidemiological Unit of Borno State Ministry of Health in April 2024. One Poliovirus serotype was handled at a time. A 75 cm^2^ culture flask was seeded with the L20B cell suspension of 1.0x 10^5^/ml concentration using the growth medium (GM), which consisted of Eagle's Minimum Essential Medium (MEM) supplemented with with 10% foetal bovine serum, 2 mM glutamine, 2% Penicillin/Streptomycin and 1 % HEPES and 2.5 % sodium bicarbonate and incubated at 36 ± 1 °C for 48 h or until 70–80 % confluency was attained. Then, 0.4 ml of the reference strain was inoculated into the flask and incubated again at 36 ± 1 °C for I hour, rotating the flask every 15 min for even distribution and adsorption of the virus onto the cells. Thereafter, 25 ml of the maintenance medium (MM) constituted similarly to GM but supplemented with with 2% foetal bovine serum, 2 mM glutamine, 2% Penicillin/Streptomycin and 1 % HEPES and 2.5 % sodium bicarbonate, was added. The flask was incubated at 36 ± 1 °C for five days with daily examination for CPE. Once the CPE was up to 100 %, the flask was harvested and freeze-thawed thrice to disintegrate the virus from the cells. The suspension was centrifuged at 3000 rpm for 30 min. O.2 ml aliquots of the local virus stocks (LVS) were stored in cryovials at −80 °C. The LVS of each serotype was confirmed using qTR-PCR as described by Gerloff et al.^29^ (Supplementary Fig. 1).

### 2.7 Validation of the local virus stock using the reference strains

The titre of each LVS was determined in parallel with the NIBSC reference strains and nOPV2 three consecutive times on different days and using fresh dilutions according to the protocol in the WHO Polio Laboratory Manual.^28^

### 2.8 Validity conditions

According to the WHO Laboratory Manual,^28^ the titers of ± 0.5 log between the LVS and Reference strains indicated the validity of the test. Titers of 5.1 logs, with values between 4.6 logs and 5.6 logs, were still considered valid. After obtaining valid titers from both the Reference/vaccine strains and LVS, the neutralization tests were performed using the LSVs.

### 2.9 Microneutralization test (MNT)

MNT against poliovirus described in the WHO Laboratory Manual,^28^ was adopted in this study. Sera were inactivated at 56 °C for 30 minutes. The L20B cell line was grown in growth medium (GM) consisting of Eagle's minimal essential medium (EMEM) supplemented with 10 % fetal bovine serum, 2 mM glutamine, penicillin/streptomycin, 1 % HEPES, and 2.5 % sodium bicarbonate. Both virus stock and serum were diluted using in‐house diluent (IHD) constituted with phosphate buffer saline supplemented with penicillin/streptomycin (22 %), gentamycin (0.2 %), and fungizone (0.02 %). Each serum was diluted in a 2-fold gradient to 1:1024, while the virus stock was diluted to contain 100 CCID50/ml. Two wells per serum dilution were used. An equal volume of each serum dilution and virus dilution was mixed and incubated for 1 h at 36 °C. A 96‐ microtiter plate was seeded with 100 μL of L20B cell suspension diluted to contain 2 × 10^5^ cells/mL using GM. The cells were incubated at 36 °C till 70 %–80 % confluence was attained before inoculation with virus/serum dilution: With the aid of a multichannel pipette, GM was carefully removed from the cell, and 100 μL of the serum-virus mixture was added to the appropriate wells and incubated at 36 ± 1 °C for 1 h. The controls used on each plate were virus controls (back titration), cell controls (neither virus nor serum), and a local in-house reference serum to standardize the protocol. The local in-house reference serum refers to pooled (3–5) positive sera for PV1, 2, and 3, with known neutralizing titers. These sera were stored at −80 °C, and the titers must be ± 0.5 from the initial titers against the LVS.

. During incubation, the plate was rotated every 15 min for even distribution of the inoculum on the cells. Thereafter, 100 μL of MM was added to each well. The plate was sealed, incubated at 36 ± 1 °C for 7 days, and examined daily for cytopathic effect (CPE). The above procedure was carried out in a BSL-2. Each serum was tested for PV type 1, 2 (nOPV2) and 3. The wells that showed 100 % CPE were considered negative and the reciprocal of the highest dilution of the serum that neutralized 100 % of the virus was the nAb titer in the sample. The MNT results were based on the visualization of CPE as an indicator of infectivity. To increase the visualization of the results, the wells were stained with crystal violet as follows: the MM was discarded from all the wells using a Pasteur pipette, and fixed with 500 μL of 10 % formaldehyde for 30 min. After discarding the formaldehyde, the wells were stained with 100 μL of 0.5 % crystal violet in ethanol for 5–10 min. The plates were washed with tap water and air-dried.

### 2.10 Research ethics

The research ethical approval letter was obtained from the Research and Ethics Committee of Borno State Ministry of Health with reference number: BMOH/19//2023 dated 29 February 2023 (Bama) and BMOH/17//2023, dated 16 February 2023 (MMC). Only the children whose parents/caregivers signed informed consent forms were recruited for the study.

### 2.11 Statistical analysis

The data was analyzed using IBM SPSS Statistical software version 25, which applied descriptive and inferential statistical methods. Descriptive statistics, including frequencies, percentages, means, and standard deviations, were used to summarize demographic variables such as gender, age, type of immunization activity, and local government area. To assess relationships and statistical significance, chi-square tests were conducted to determine associations between categorical variables. Logistic regression was used to predict the probability of having neutralizing antibodies based on age, gender, and vaccine doses.

Analysis of variance (ANOVA) was applied to compare mean antibody titers across different age groups, gender, and vaccine doses. Additionally, Poisson regression was used to model the count data of positive antibody titers, and survival analysis through the Cox proportional-hazards model examined the time taken for children to achieve a certain antibody titer level based on demographic and vaccination factors. Correlation analysis assessed the strength and direction of the relationship between the number of vaccine doses and antibody titers.

Further analysis involved clustering techniques, where K-means clustering categorized children into groups based on their antibody titers, identifying clusters with high, moderate, and low immunity. Decision tree analysis classified antibody status based on age and number of vaccine doses, offering insights into how different factors influence immunity. Finally, 95 % confidence intervals were computed to estimate the range within which the true proportion of children with antibodies is likely to fall for each poliovirus serotype, providing a more precise understanding of immunity distribution in the study population.

## 3 Results

### 3.1 The demographic characteristics of the study population

A slightly higher proportion of males (56.0 %) compared to females (44.0 %) was studied. The majority (28.3 %) of the subjects were in the 7–9 years age group, which is above the target age (0–5 years) for polio vaccination campaigns, with notable representation from the 1–3 years age group (22.3 %) (Table 1). The majority of subjects were part of Supplementary Immunization Activities (SIA) at 72.3 %, with a smaller proportion involved in Routine Immunization (RI) at 15.3 %. A mix of Routine and Supplementary Immunization (RT/SIA) reflects ongoing and comprehensive vaccination efforts (Supplementary Table 1).

### 3.2 Children with neutralizing antibody to either one or a combination of poliovirus serotypes among the study population

Of 400 children tested, 309 (77.3 %), 253 (63.3 %), and 308 (77.0 %) had nAb against individual Poliovirus serotypes 1, 2, and 3, respectively. However, 191 (47.8 %) had nAb against the combination of Poliovirus serotypes 1, 2, and 3 simultaneously. 213 (53.3 %) had nAb to a combination of P1P2, 269 (67.3 %) against P1P3, and 213 (53.3 %) against P2P3 (Table 2).

**Table 2:** Percentage of children with neutralizing antibody to either one or combination of poliovirus serotypes among the study population.

Description	No. of children with nAb	Children with nAb (%)	Interval
Positives for P1	309	77.3	
Positives for P2	253	63.3	0.589-0.677
Positives for P3	308	77.0	0.724-0.804
Positives for P1, P2, and P3	191	47.8	
Positives for P1 and P2	213	53.3	
Positives for P1 and P3	269	67.3	
Positives for P2 and P3	213	53.3	

Of 400 children tested, 309 (77.3 %), 253 (63.3 %), and 308 (77.0 %) had nAb against individual Poliovirus serotypes 1, 2, and 3 respectively. However, 191 (47.8 %) had nAb against the combination of Poliovirus serotypes 1, 2, and 3 simultaneously. 213 (53.3 %) had nAb to a combination of P1P2, 269 (67.3 %) against P1P3, and 213 (53.3 %) against P2P3.

### 3.3 Immunity gaps for specific poliovirus serotypes based on the presence of neutralizing antibodies

Of 400 children studied, 91 (22.8 %) had no nAb against P1 but were protected against P2 (44.0 %) and P3 (42.9 %). 147 (36.8 %) children who had no nAb against P2, however, had it against P1 (65.3 %) and P3 (64.6 %). 92(23.0 %) who had nAb against P1 (43.5 %) and P2 (43.5 %) had none against P3 (23.0 %). Those (12.8 %) who had no nAb against the combination of P1P2 were protected against P3 (33.3 %). Children with no nAb against the combination of P1P3 and P2P3 had it against P2 (34.6 %) and P1 (34 %), respectively (Table 3).

**Table 3:** Children who lacked neutralizing antibody for one serotype but are protected against other serotypes.

Poliovirus serotypes	Number of children without neutralizing antibody (%)	Number of children with neutralizing antibodies to other serotypes	
P1	91 (22.8)	P2 = 40 (44.0)	P3 = 39 (42.9)
P2	147 (36.8)	P1 = 96 (65.3)	P3 = 95 (64.6)
P3	92 (23.0)	P1 = 40 (43.5)	P2 = 40 (43.5)
P1P2	51 (12.8)	P3 = 17 (33.3)	NA
P1 P3	52 (13.0)	P2 = 18 (34.6)	NA
P2P3	52 (13.0)	P1 = 18 (34.6)	NA

Of 400 children studied, 91(22.8 %) had no nAb against P1 but were protected against P2 (44.0 %) and P3 (42.9 %). 147 (36.8 %) children who had no nAb against P2, however, had it against P1 (65.3 %) and P3 (64.6 %). 92(23.0 %) who had nAb against P1 (43.5 %) and P2 (43.5 %) had none against P3 (23.0 %). Those (12.8 %) who had no nAb against the combination of P1P2 were protected against P3 (33.3 %). Children with no nAb against the combination of P1P3 and P2P3 had it against P2 (34.6 %) and P1 (34 %), respectively.

### 3.4 Gender distribution of neutralizing antibodies to either single or a combination of poliovirus serotypes

**Fig. 2:**
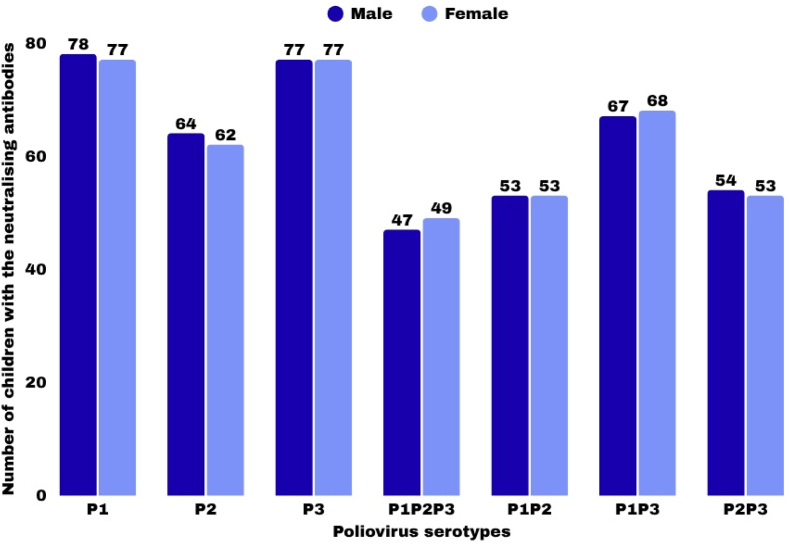
Sex distribution of neutralizing antibody to either single or combination of poliovirus serotypes. Among the male participants, 78 % had neutralizing antibodies against P1, compared to 77 % of females. 62 % females and 64 % males had nAb against P2. However, 77 % of males and 77 % females were protected against P3. The presence of nAb against the combination of P1P2P3 was observed among 47 % of males and 49 % of females. Protection against the combination of P1P2 was found in 53 % of males and 53 % of females, while protection against P1P3 was observed in 67 % of males and 68 % of females. Both sexes (54 % males and 53 % females) had nAb against P2P3.

Among male participants, 78 % had neutralizing antibodies against P1, compared with 62 % of females. More females (77 %) had nAb against P2 than males (64 %). However, 76 % of males and 49 % females were protected against P3. The presence of nAb against the combination of P1P2P3 was observed among 47 % of males and 53 % of females. Protection against the combination of P1P2 was found in 54 % of males and 94 % of females, while P1P3 was observed in 67 % of males and 68 % of females. Both sexes (54 % males and 52 % females) had nAb against P2P3. With Chi-square analysis, the sex of the study population had no significant effect on the presence of neutralizing antibodies (Fig. 2). However, with the ANOVA test, the mean antibody titers and gender were significantly different (F = 8.35, p = 0.004). Thus, gender-specific factors may influence antibody responses, information that could be relevant for designing more effective vaccination strategies. The Cox Proportional-Hazards model suggests that gender differences could favor the speed at which children achieve the target antibody titers with males having a higher hazard ratio than females.

### 3.5 The age distribution of neutralizing antibodies against a single or a combination of poliovirus serotypes

All age groups studied had significant neutralizing antibodies against the different serotypes of PV. Overall, the lowest percentage of children protected against PV was in the 1–3 years group. The highest proportion of protected children against serotype 2 was among children aged 7–12 years. The children with nAb to the combination of the three serotypes (P1P2P3) were in the 7–12 years group. The chi-square test revealed that the effectiveness of the polio vaccine in inducing neutralizing antibodies does not vary significantly with age. However, the ANOVA test showed that the mean antibody titers for all three poliovirus serotypes varied across the different age groups. Similarly, the Cox Proportional-Hazards model suggests that an increase in age increases the likelihood of achieving the desired antibody titer within a given time frame.

### 3.6 Distribution of neutralizing antibodies to either one or a combination of poliovirus serotypes and the doses of poliovirus vaccines

**Fig. 3:**
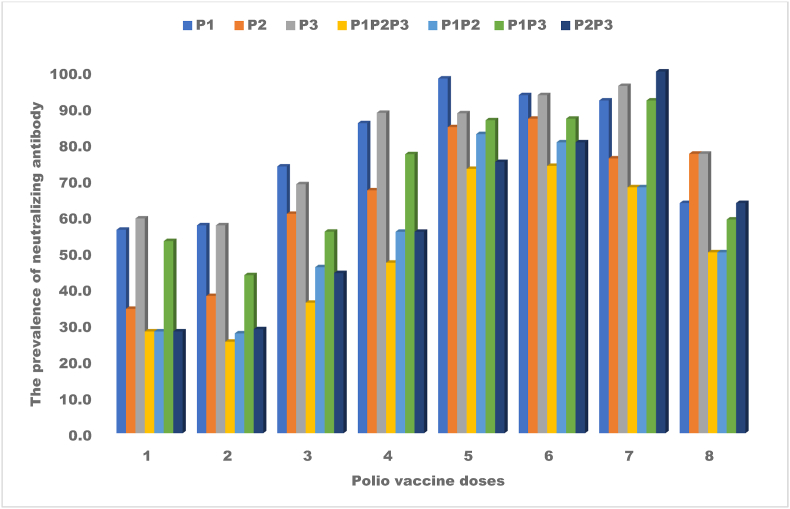
Distribution of neutralizing antibodies to either one or a combination of poliovirus serotypes and the doses of poliovirus vaccines All participants received nOPV2 in 2022 through SIA (Outbreak response) (Supplementary Table 2), while bOPV and IPV were administered during RI. The proportion of children with neutralizing antibodies against individual serotypes of PV (P1, P2, and P3) increased with the number of vaccine doses (received through OBR) till over 8, after which the seroprevalence rates declined (Fig. 3). A similar trend was obtained for P1P2, P1P3, and P2P3, demonstrating the cumulative effect of additional doses on achieving immunity to either a single or a combination of PV serotypes.

All participants received nOPV2 in 2022 through SIA/Outbreak response (Supplementary Table 2), while bOPV and IPV were administered during RI. The proportion of children with neutralizing antibodies against individual serotypes of PV (P1, P2, and P3) increased with the number of vaccine doses (received through SIA/OBR) till over 8, after which the seroprevalence rates declined (Fig. 3). A similar trend was obtained for P1P2, P1P3, and P2P3, demonstrating the cumulative effect of additional doses on achieving immunity to either a single or a combination of PV serotypes. For doses 2, 3, 5, 6, 7, and 8, the p-values were above 0.05, showing no significant relationship between the dose of the polio vaccine and the presence of neutralizing antibodies for P1. However, a significant association was observed between doses 1 (p = 0.042) and 4 (p = 0.024) of the vaccine and nAb against P2, while doses 2, 3, 5, 6, 7, and 8, showed no significant relationships (p>) 0.05. An ANOVA revealed a significant difference between mean nAb titers for poliovirus serotypes 1, 2, and 3 across varying vaccine doses (F-value = 12.50, p = 0.001). The correlation coefficients between the number of doses and antibody titers for each poliovirus serotype range from 0.35 to 0.65 for P1, 0.40 to 0.58 for P2, and 0.45 to 0.65 for P3. These increasing correlations suggest a positive relationship between the number of doses and the antibody titers.

## 4 Distribution of neutralizing antibodies by immunization activities

**Fig. 4:**
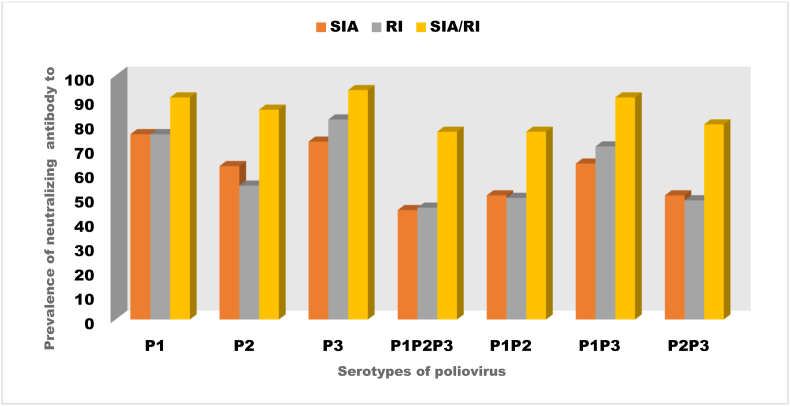
The distribution of neutralizing antibodies to different serotypes of Poliovirus according to the type of immunization activities The majority of the children tested (72.3 %) received the Polio vaccine through Supplementary Immunization Activities (SIA), while 19 % got it through routine immunization (RI). The minority (8.8 %) received the vaccine through the SIA and RI (Fig. 4). The Presence of nAb against either a single PV serotype or their combinations were much higher among those who received the Polio vaccine through a combination of SIA and RI.

The majority of the children tested (72.3 %) received the polio vaccine through Supplementary Immunization activities (SIA)/OBR, while 19 % got it through routine immunization (RI). The minority (8.8 %) received the vaccine through the SIA and RI (Fig. 4). The Presence of nAb against either a single PV serotype or combinations of all the serotypes was much higher among those who received the polio vaccine through a combination of SIA and RI. Therefore, combining these strategies appears to produce a robust immune response across all poliovirus serotypes. Overall, this strategy indicated enhanced effectiveness in achieving higher antibody levels.

### 4.1 Neutralizing antibody titers for the three serotypes of poliovirus

At a dilution of 1:10, 50 children had detectable nAb for P1. This number increased to 61 at 1:20, peaked to 79 at 1:40, and decreased at higher dilutions. At the highest dilution of 1:10240, no individual had a detectable antibody for P1. Children with nAb against P2 increased from 32 at dilution 1:10 to 60 at 1:20, and peaked to 80 at 1:40 and thereafter declined at higher dilutions. Only 4 children had nAb at a dilution of 1:1280 but none at 1:5120. At a dilution of 1:10, 24 children had detectable nAb against P3, increased to 58 at 1:80, and thereafter declined gradually at higher dilutions. At the highest dilution of 1:10240, 6 individuals had detectable nAb for P3 (Table 5).

## 5 Discussion

Achieving population immunity of 80 % against the three serotypes of PV simultaneously is the global target of GPEI. Only 48 % achieved that target in this study (Table 2). This seroprevalence rate in this study was lower than the 53 % obtained in a similar study in the same environment in 2012^30^ , and 64.3 % in South Western Nigeria.^31^ The difference in these rates could be attributed to the type of polio vaccine used during campaigns in the previous (tOPV) and the current studies (bOPV and nOPV2). Also, in 2012 and 2015, Nigeria was still endemic for both WPV and VDPV, unlike in 2023 (when samples for this study were collected), with only the circulation of cVDPVs. We speculate that the acceptance of the poliovirus vaccine has declined after the country successfully interrupted the transmission of the WPV in August 2020. It is envisaged that the findings of this study will serve as an eye-opener and a wake-up call to GPEI to intensify efforts towards the targeted population immunity.

In this study, with a reasonable degree of certainty, high immunity levels were observed for the individual poliovirus serotype 1 (77.3 %) and 3 (76.5 %), but lower for 2 (63.3 %) (Table 2), indicating a seroconversion pattern of P1> P3> P2 (Table 2) in contrast to P2> P1> P3 reported in South Western Nigeria,^31^^,^^32^ China,^33^ India^34^ and DRC.^35^ In 2012, the P1>P2 & P3 pattern was obtained from Borno,^24^ P1> P3 > P2 in Zaria,^36^ while P1>P2>P3 was demonstrated in Kano State, Nigeria^37^ and Italy 39]. The actual reasons for the disparity in seroconversion patterns are not known, but environmental and genetic factors from the vaccinees could be contributory, in agreement with previous reports.^38^^,^^39^. Overall, this study has further confirmed the disparity in immunity to PV infections between and within communities in Nigeria and other countries.^31^^,^^40^ Notably, pre-existing immunity to one serotype cannot impact or affect seroconversion to the other two serotypes.^41^

Additionally, the level of immunity gaps against different serotypes of poliovirus was observed among the vaccinated children. For instance, this study revealed that 91 (22.8 %) vaccinated children lacked nAb against P1, 147 (36.8 %) against P2, and 94 (23.0 %) against P3, while 34 (8.5 %) had no nAb against the three serotypes (Table 3). Although immunity gaps differed, the proportion of children lacking protection against either of the three PV serotypes can initiate epidemics if WPV is imported into such communities. Specifically, the higher immunity gap for P2 suggests that many children were missed during the nOPV2 outbreak response SIAs in Borno State in 2023 (Table 3 & Supplementary Table 2). It has also indicated a substantially greater risk for further spread of cVDPV2 outbreaks, in agreement with a previous report.^42^ Although the gaps for P1 and P3 are lower, they still represent a segment of the vaccinated population that remains unprotected and is at risk of reinfection by either WPV/VDPV 1 and 3, in agreement with a previous report.^8^ The immunity gap for P1 and P3 could be attributed to the lack of bOPV SIA in Borno State in 2023 (Supplementary Table 2). We suggest improved bOPV and fIPV in RI in parallel with high-quality bOPV SIAs and nOPV2 SIAs to prevent the resurgence of poliovirus and ensure comprehensive protection against the disease. Furthermore, our findings underscore the need for continued monitoring of polio vaccine seroconversion rates for prompt identification of emerging gaps in immunity. Such a revelation will guide and direct judicious targeting of limited resources to foster protection. The seroconversion pattern of polio vaccine to the three serotypes of poliovirus in children and adults was comparable. Thirty staff of the WHO National Polio Laboratory, whose ages ranged from 21 to 70 years (Data not included), received tOPV in 2016 and were assessed for neutralizing antibodies to the three serotypes of poliovirus in 2024. The results showed that only 52 % had nAb against the P1P2P3 simultaneously, while 9.7 %, 26 %, and 29 % lacked immunity against P1, P2, and P3, respectively. The identified staff with immunity gaps were revaccinated with IPV and retested 12 months post-vaccination. Interestingly, their nAb titers increased significantly (≥4 fold). Incidentally, a staff member who did not receive the tOPV initially, along with others, but received IPV for the first time, had a very low nAb titer (P1 = 1:8, P2 = 1:16, and P3 = 1:4). This experience shows that both children and adults respond differently to each of the serotypes in the polio vaccine. Additionally, subjects primed with OPV and boosted using IPV seem to increase the seroconversion rates significantly, in agreement with the previous reports.^43^^,^^44^

In this study, the effectiveness of the polio vaccine in inducing neutralizing antibodies does not vary significantly with age (Table 4) and gender (p > 0.05) (Fig. 2), in agreement with a previous study,^30^ but in contrast to Bolu et al..^8^ Although some studies have reported no significant differences in antibody levels between the ages of the polio vaccine recipients^45^^,^^46^ and gender,^32^ others have demonstrated variations in the antibody titers and GMTs with age^47^^,^^48^ and gender.^46^ Notably, other factors, including vaccination,^49^^,^^50^ exposure to wild poliovirus (52), and other health conditions,^51^ can also influence antibody responses.

**Table 4:** The age distribution of children with neutralizing antibodies against a single or combination of poliovirus serotypes.

Age (years)	Total No. Tested	No. Positive						
		P1 (%)	P2 (%)	P3 (%)	P1P2P3(%)	P1P2(%)	P1P3(%)	P2P3(%)
1–3	89	55 (62)	45 (51)	51 (57)	32 (36)	35 (39)	43 (48)	36 (40)
4–6	82	67 (82)	52 (63)	66 (80)	38 (46)	46 (56)	57 (70)	42 (51)
7–9	114	95 (83)	81 (71)	92 (72)	64 (56)	71 (62)	85 (75)	69 (61)
10–12	67	56 (84)	47 (70)	55 (82)	38 (57)	40 (60)	51 (76)	42 (63)
13–15	48	36 (75)	28 (58)	42 (58)	20 (42)	21 (44)	34 (71)	24 (50)
Total	400	309 (77.3)	253 (63.3)	306 (76.5)	192 (48.0)	213 (53.3)	270 (67.5)	213 (53.2)

All age groups studied had significant neutralizing antibodies against the different serotypes of PV. Overall, the lowest percentage of children protected against PV was in the 1–3 years group. The highest proportion of protected children against serotype 2 was among children aged 7–12 years. The children with nAb to the combination of the three serotypes (P1P2P3) were in the 7–12 years group.

**Table 5:** Neutralizing antibody titers for the three serotypes of Poliovirus.

Neutralizing antibody titers	P1	P2	P3
1:10	50	32	24
1:20	61	60	46
1:40	79	80	52
1:80	62	45	58
1:160	29	17	51
1:320	13	8	24
1:640	8	5	10
1:1280	1	4	5
1: 2560	4	2	18
1; 5120	2	0	12
1:10240	0	0	6
Total	309	253	306

At a dilution of 1:10, 50 children had detectable nAb for P1. This number increased to 61 at 1:20, peaked to 79 at 1:40, and decreased at higher dilutions. At the highest dilution of 1:10240, no individual had a detectable antibody for P1. Children with nAb against P2 increased from 32 at dilution 1:10 to 60 at 1:20, and peaked to 80 at 1:40 and thereafter declined at higher dilutions. Only 4 children had nAb at a dilution of 1:1280 but none at 1:5120. At a dilution of 1:10, 24 children had detectable nAb against P3, increased to 58 at 1:80, and thereafter declined gradually at higher dilutions. At the highest dilution of 1:10240, 6 individuals had detectable nAb for P3.

This study also revealed that the mean antibody titers for the three poliovirus serotypes varied significantly across the different age groups, in agreement with a previous report.^31^ This implies that the likelihood of achieving the desired antibody titer within a given time frame may increase with age. Furthermore, the mean antibody titers and gender were significantly different (F = 8.35, p = 0.004), implying that gender-specific factors may influence antibody responses. Further analysis suggests that gender differences may favor the speed at which children achieve the target antibody titers.

This study revealed that the relationship between the number of vaccine doses and the presence of nAb differed across different poliovirus serotypes (Fig. 3). Four doses of the vaccine were significantly associated with the presence of antibodies against serotypes 2 (p = 0.024) and 3 (p = 0.027), and only 1 dose for serotype 2 (p = 0.042). This implies that increasing the number of doses can enhance the likelihood of nAb against P2 and P3, and only one dose of nOPV2 can induce nAb. However, a significant difference was observed between mean nAb titers for poliovirus serotypes P1, P2, and P3 across varying vaccine doses (F-value = 12.50, p = 0.001). This study also revealed that not all doses of the polio vaccine produced significant effects, suggesting that the optimal number of doses for effective seroconversion might vary the serotype. Additionally, younger children (<6 years) who had received four or more polio vaccine doses had high levels of nAb. However, older children (>6 years) who received fewer doses of the polio vaccine had lower nAb than the root node group, and younger children with fewer doses the lowest levels of nAb.

The percentage of children (12.2 %) who had an nAb titer of <1:10 against the three serotypes (Table 5) was higher in this study (Borno State) than in a previous report (Ogun State -Southwestern Nigeria), with only 3.7 %^31^, and 5.1 % among adults in Brazil.^52^ This category of subjects could be re-infected with the wild or mutated vaccine strain of the virus.^53^ However, if that happens, such children may not develop paralytic polio but could serve as sources of infection to the immunologically naïve subjects.^54^

The majority of the children tested (72.3 %) received the polio vaccine through SIAs, with a smaller proportion (15.3 %) from Routine Immunization (RI) and a minority (8.8 %) through a combination of SIA and RI (Fig. 4). The type of immunization strategies through which the children received the polio vaccine and nAb were significantly associated with P2 and P3 (but not P1. This finding suggests that the polio vaccine administered through certain immunization strategies may elicit a more robust immune response against P2 and P3 than against P1. This could be due to the variations in vaccine efficacy or differences in how a child is exposed to the polio vaccine in each of the strategies (Supplementary Table 2). Further investigation may be needed to determine which immunization strategy provides the best immunity and whether adjustments to vaccination programs are required to enhance immunity against all three poliovirus serotypes.

The limitations of this study include our inability to differentiate antibodies induced by the polio vaccine and natural infections. Additionally, the doses considered in this study did not include those received in the previous years before sample collection in 2023.

## 6 Conclusion

High immunity levels were observed for P1 and P3, but lower for P2. Despite several cVDPV outbreak response campaigns conducted with nOPV2, the population immunity against the combination of P1P2P3 is still low (48 %). Some pockets of children still lacked immunity against P1 and P3. The mean antibody titers for all three poliovirus serotypes varied significantly across the different age groups. Similarly, gender differences may influence the speed at which children achieve the target antibody titers, with males having a higher hazard ratio than females. The number of vaccine doses and the presence of nAb varied across different poliovirus serotypes. The type of immunization strategies through which the children received the polio vaccine and nAb was significantly associated. The administrative data are not commensurate with population immunity. New strategies to improve RI coverage and immunity against the three serotypes should be implemented urgently.

## CRediT authorship contribution statement

**Marycelin Mandu Baba:** Writing – review & editing, Writing – original draft, Formal analysis, Conceptualization. **Aisha Abba Kawu:** Methodology, Investigation. **Sadiya Alhaji Bukar:** Methodology, Investigation. **Musa Sundu Melton:** Writing – original draft, Data curation. **Abdulwahab Mala:** Writing – review & editing, Validation, Data curation. **Ibrahim Salisu:** Project administration, Data curation. **Bamidele Soji Oderinde:** Writing – original draft, Project administration, Methodology.

## Funding

This research did not receive any specific grant from funding agencies in the public, commercial, or not-for-profit sectors.

## Declaration of competing interest

The authors declare that they have no known competing financial interests or personal relationships that could have appeared to influence the work reported in this paper.

## Data Availability

Data will be made available on request.

## Abbreviations

OPV: Oral polio vaccine, IPV: Inactivated polio vaccine, fIPV: Fractional Inactivated polio vaccine, bOPV: bivalent Oral polio vaccine (serotype 1 and 3), tOPV: trivalent oral polio vaccine (serotypes 1, 2,3 and), nOPV2: novel oral Polio vaccine, WHO: World Health Organization, GPEI: Global Polio Eradication Initiative, cVDPV: circulating vaccine-derived poliovirus, AFP: Acute flaccid paralysis, MMC: Maiduguri Metropolitan Council, LGA: Local government area, IDP: Internally displaced person, NISSC: National Institute of Biological Standards, UK: United Kingdom, LVS: Local virus stock, GM: Growth medium, MEM: Minimum essential medium, L20B: a mouse cell line genetically engineered to express the human poliovirus receptor CD155 m CC: Cell culture control, MNT: Microneutralization test, IHD: In-house diluent, CCID_50_: Cell culture infectious dose 50, PV: poliovirus, CPE: Cytopathic effect, ANOVA: Analysis of variance, SIA: Supplementary Immunization activities, P1: poliovirus serotype1, P2: poliovirus serotype2, P3: poliovirus serotype 3, P1P2P3: Combination poliovirus serotypes 1,2 and 3, P1P2: combination poliovirus serotypes 1and 2, P1P3: combination poliovirus serotypes 1 and 3, P2P3: combination poliovirus serotypes 2 and 3, nAb: neutralizing antibody, RI: Routine immunization.
