# Supplementary figures and images for: Population immunity to the three serotypes of poliovirus post-interruption of wild poliovirus transmission in Nigeria
Source: J Virus Erad. 2025 Oct 29;11(4):100615. doi: 10.1016/j.jve.2025.100615 (PMC12648957; doi:10.1016/j.jve.2025.100615)

**Supplementary figure 1: Pictures of L20B cell line with CPE and uninoculated cells**


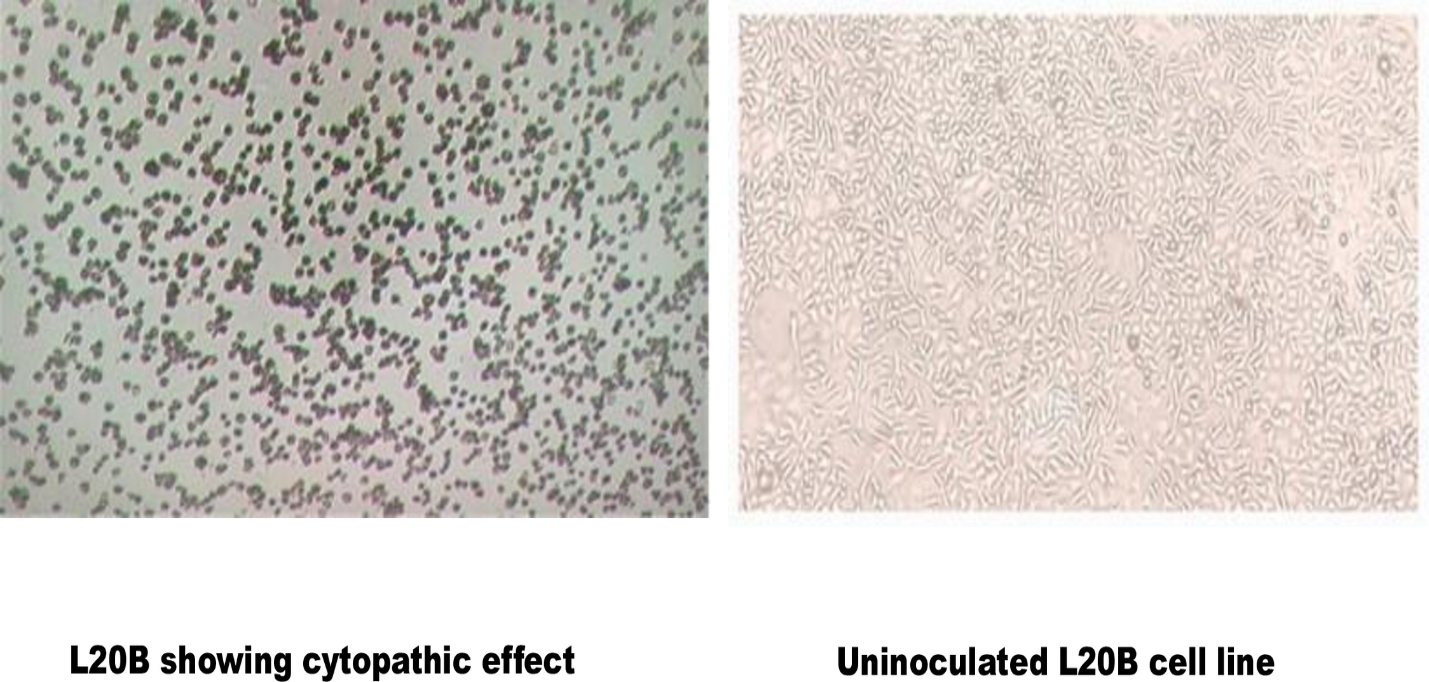

Supplement: Multimedia component 1 [file mmc1.docx]

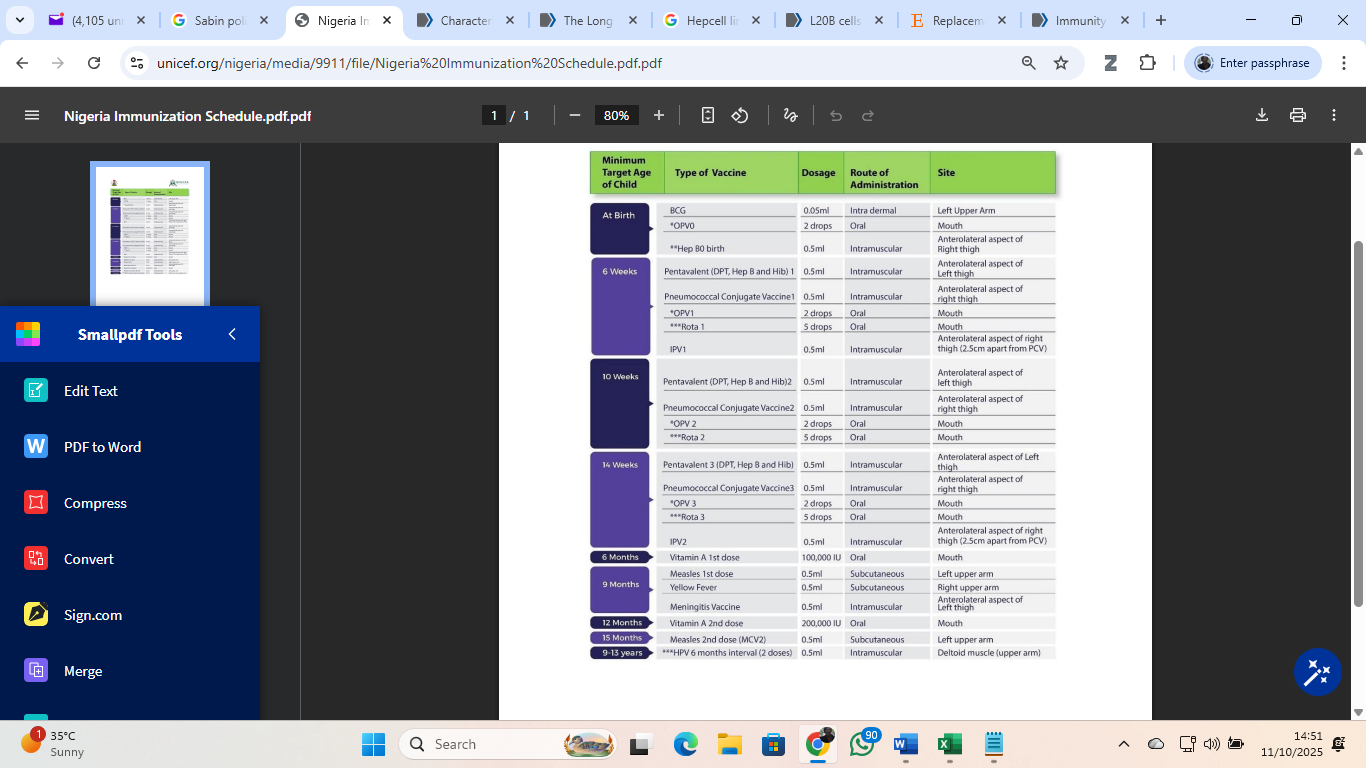

Supplement: Multimedia component 5 [file mmc5.docx]
