# Supplementary material for: Population immunity to the three serotypes of poliovirus post-interruption of wild poliovirus transmission in Nigeria
Source: J Virus Erad. 2025 Oct 29;11(4):100615. doi: 10.1016/j.jve.2025.100615 (PMC12648957; doi:10.1016/j.jve.2025.100615)
Supplement: Multimedia component 3 [file mmc3.docx]

| **S/N** | **Campaign type** | **Vaccine type** |  | **%Coverage(nOPV)** | **% coverage (fIPV)** |
| --- | --- | --- | --- | --- | --- |
| 1 | July 2023 | nOPV 2 | OBR | 102 | Nil |
| 2 | August 2023 | nOPV2 | OBR | 102 | 99 |
| 3 | November 2023 | nOPV2+fIPV | OBR | 100 | Nil |
| 4 | Dec 2023 | nOPV2 | OBR | 95 | Nil |

**2023 Borno State OBRs (SIAs)**

The outbreak response activities conducted in Borno State in 2023
