# Supplementary material for: Population immunity to the three serotypes of poliovirus post-interruption of wild poliovirus transmission in Nigeria
Source: J Virus Erad. 2025 Oct 29;11(4):100615. doi: 10.1016/j.jve.2025.100615 (PMC12648957; doi:10.1016/j.jve.2025.100615)
Supplement: Multimedia component 4 [file mmc4.docx]

**Supplementary Table 3**

**Chi-Square Test Results for the Presence of Neutralizing Antibodies by Gender**

| **Gender** | **Serotype** | **Chi-Square Statistic** | **Degrees of Freedom** | **p-Value** |
| --- | --- | --- | --- | --- |
| Male | P1 | 0.92 | 1 | 0.34 |
| Female | P1 | 0.92 | 1 | 0.34 |
| Male | P2 | 1.21 | 1 | 0.27 |
| Female | P2 | 1.21 | 1 | 0.27 |
| Male | P3 | 0.15 | 1 | 0.70 |
| Female | P3 | 0.15 | 1 | 0.70 |

**ANOVA Table for difference in the mean antibody titres for Poliovirus serotypes P1, P2, and P3 across gender**

| **Source of Variation** | **SS** | **df** | **MS** | **F** | **p-value** |
| --- | --- | --- | --- | --- | --- |
| Between Groups (Gender) | 1,000 | 1 | 1,000 | 8.45 | 0.004 |
| Within Groups (Error) | 47,500 | 398 | 119.3 |  |  |
| **Total** | **48,500** | **399** |  |  |  |
